# Molecular Tunnels in Enzymes and Thermophily: A Case Study on the Relationship to Growth Temperature

**DOI:** 10.3390/microorganisms6040109

**Published:** 2018-10-20

**Authors:** Juan Miguel Gonzalez

**Affiliations:** Instituto de Recursos Naturales y Agrobiología, Consejo Superior de Investigaciones Científicas, IRNAS-CSIC, Avda. Reina Mercedes 10, 41702 Sevilla, Spain; jmgrau@irnase.csic.es; Tel.: +34-95-462-4711; Fax: +34-95-462-4002

**Keywords:** molecular tunnels, enzyme structure, enzyme activity, temperature, thermophile, thermophily, enzyme thermostability

## Abstract

Developments in protein expression, analysis and computational capabilities are decisively contributing to a better understanding of the structure of proteins and their relationship to function. Proteins are known to be adapted to the growth rate of microorganisms and some microorganisms (named (hyper)thermophiles) thrive optimally at high temperatures, even above 100 °C. Nevertheless, some biomolecules show great instability at high temperatures and some of them are universal and required substrates and cofactors in multiple enzymatic reactions for all (both mesophiles and thermophiles) living cells. Only a few possibilities have been pointed out to explain the mechanisms that thermophiles use to successfully thrive under high temperatures. As one of these alternatives, the role of molecular tunnels or channels in enzymes has been suggested but remains to be elucidated. This study presents an analysis of channels in proteins (i.e., substrate tunnels), comparing two different protein types, glutamate dehydrogenase and glutamine phosphoribosylpyrophosphate amidotransferase, which are supposed to present a different strategy on the requirement for substrate tunnels with low and high needs for tunneling, respectively. The search and comparison of molecular tunnels in these proteins from microorganisms thriving optimally from 15 °C to 100 °C suggested that those tunnels in (hyper)thermophiles are required and optimized to specific dimensions at high temperatures for the enzyme glutamine phosphoribosylpyrophosphate amidotransferase. For the enzyme glutamate dehydrogenase, a reduction of empty spaces within the protein could explain the optimization at increasing temperatures. This analysis provides further evidence on molecular channeling as a feasible mechanism in hyperthermophiles with multiple relevant consequences contributing to better understand how they live under those extreme conditions.

## 1. Introduction

Biomacromolecular channels (tunnels and pores) present critical structural importance which is intimately related to the biological function and structural stability of macromolecules [1,2,3]. The access of substrates to the active sites of enzymes occurs in many cases through tunnels that connect the exterior to the internal protein spaces and in a reverse way for the products of biological reactions. In addition, tunnels have been shown to form internal passages between active sites and protein subunits leading to a quick interconnection between proteins. In this way, specific substrates can be transferred, increasing stability and accelerating reaction times [4]. Channels (at least 15 Å long) have been reported in 64% of enzymes with known crystal structures [5], suggesting that they are common features for the structure of many enzymes and relevant to their functionality.

Molecular channeling has been proposed to have great importance in various aspects of protein structure and enzyme activity [6,7]. For instance, molecular channeling reduces the time required for the transit of reaction intermediates between active centers of different enzymes, or within multifunctional enzymes, connecting one active site to another. The mechanism of substrate tunneling contributes to minimize the loss of reaction intermediates by diffusion contributing to increase enzyme efficiency. Another advantage of tunneling is to protect labile reaction intermediates that otherwise would be rapidly degraded by exposure to solvents or high temperatures. In addition, the isolation through molecular channels of substrates, products or intermediates could avoid potential inhibitory effects to the cell and to other enzymes. Additionally, using tunnels, substrates, and reaction intermediates, can be preserved from their use in other distinct metabolic pathways, diminishing competition and avoiding a potential unfavorable equilibrium. On top of these numerous features, molecular tunneling could limit the use of substrates, intermediates and products to a specific multi-step chain of related enzymes within a pathway, in order to increase metabolic efficiency and accelerate the stepping-stone progress of a sequential catalytic transformation. Although the potential importance of molecular tunnels has been shown [5,6,7], the relevance of this process on the ability of (hyper)thermophiles to thrive under high temperatures remains clearly understudied.

There are numerous studies explaining the possibilities and different alternatives so that nucleic acids, proteins and lipids can maintain their stability in cells living at high temperatures [8,9,10]. Nevertheless, a gap in knowledge remains on how thermophiles can thrive under high temperatures using mostly the same low-molecular weight, thermolabile, and biomolecules that all living cells possess. Examples of thermolabile biomolecules are ATP, NAD/H, NADP/H, and pyridoxal phosphate, among others, many of which are universal and essential for the central metabolism of all cells. Reports early this century [11,12] pointed out that several low-molecular weight biomolecules are thermolabile and their turnover rate at the growth temperatures of (hyper)thermophiles was not permissive as an efficient living alternative. This is an unresolved question, arising since the discovery of hyperthermophiles during the last half of the previous century [13,14]. Recently, Cuecas et al. [15] presented a simple mechanism that could allow thermophiles to maintain the thermal stability of some low molecular weight biomolecules such as NADH. Cuecas et al. [15] proposed that by maintaining the intracellular viscosity, those thermolabile biomolecules could be greatly stabilized. Nevertheless, this mechanism appeared to be inefficient at >80 °C, i.e., for hyperthermophiles, because of the great reduction of viscosity in aqueous solutions at temperatures close to the boiling point of water. Other complementary possibilities for the maintenance of the thermal stability of low-molecular weight biomolecules have been suggested [11,12,15] but the most likely alternative is the occurrence of molecular channeling, so that the low-molecular weight thermolabile biomolecules could be transferred directly between enzymes and maintained within molecular channels or tunnels, such that their thermal stability is preserved much longer than we would typically believe from experimentation in standard diluted aqueous solutions. However, technical and experimental difficulties have limited demonstrations of the relevance of molecular channeling and substrate tunnels in hyperthermophiles. A first step to provide evidence on the relevance of molecular channeling could be to show that the extension and volume of molecular tunnels are somehow governed by the optimum growth temperature of the microorganisms, mainly for hyperthermophiles.

With this aim in mind, this study attempts to show if there is a dependency of molecular tunnels in enzymes as a function of the optimum growth temperature for the microorganisms, with a focus mainly on (hyper)thermophiles. This analysis was comparatively performed on a range of microorganisms with optimum growth temperatures ranging from 15 °C to 100 °C, and on two enzymes, glutamate dehydrogenase (GDH) and glutamine phosphoribosylpyrophosphate amidotransferase (GPAT). GDH is a major constituent of the total protein content in hyperthermophiles (up to 20% of total protein in the hyperthermophilic archaeon *Pyrococcus furiosus* [16]; but it has not been reported to be related to molecular tunnels. GPAT has been long reported as a clear example of substrate channeling in enzymes [1,4,6,7,17]. It is hypothesized that the dimensions of the molecular tunnels in GPAT should be maintained in hyperthermophiles, although this could not be the case for GDH in hyperthermophiles, assuming enzymes need to be optimized to properly function at high temperatures.

## 2. Materials and Methods

Two case studies were analyzed, an enzyme frequently reported to present substrate tunnels of functional importance, the glutamine phosphoribosylpyrophosphate amidotransferase (GPAT; [1,4,6,7]), and an enzyme of major relevance to cells not reported to present functionally important substrate tunnels, glutamate dehydrogenase (GDH; [16,18]). Amino acid sequences for the studied enzymes covering most of the growth temperature range for microorganisms were obtained from NCBI (National Center for Biotechnology Information; https://www.ncbi.nlm.nih.gov/), and their 3D-structures were predicted by Phyre2 [19]. For GPAT, 129 and 110 3D-structures were analyzed from bacteria (109 sequences) and archaea (21 sequences), respectively. For GDH, a total of 130 3D-structures were analyzed, including bacteria and archaea. These sequences corresponded to prokaryotes with optimum growth temperatures from 15 °C to 100 °C (Supplementary Material). From the predicted structures, the tunnels in each protein were identified and localized by using the software MOLE [20]. According to Petrek et al. [20], MOLE presents a strategy for exploring molecular voids based on Voronoi diagrams, which overcomes some of the limitations (excessive computer demands and errors due to grid extrapolation) from previous software. From MOLE’s results, the total tunnel length, lateral surface and volume were calculated. The total distance or length, lateral surface and volume of the tunnels in a protein were the sums of each one of the sections identified and localized in that protein. For each section of the tunnels, lateral surface (S) and volume (V) were calculated assuming the shape of a cylinder or truncated cone using the following formulas, where r_1_ and r_2_ are the two radii of the truncated cone and h represents its height.

$$V=(1/3)\pi h\;\left(r_{1}{}^{2}+r_{1}r_{2}+r_{2}{}^{2}\right)$$

$$S=\pi \;\left(r_{1}{+r}_{2}\right)\;\sqrt{{\left(r_{1}{-r}_{2}\right)}^{2}+h^{2}}$$

Available model structures from GDH and GPAT were included in the analyses for comparative purposes against the results from predicted structures. These model structures were obtained from the Protein Data Bank (PDB; http://www.rcsb.org). For this study, only one subunit was considered so that their results are comparable to the GPAT and GDH predicted structures. A list of the model structures included in this study is available in the supplementary material, together with their PDB accession number, optimum growth temperature and bacterial species. The upper and lower limits of distribution that correspond to maximum and minimum tunnel dimensions at increasing temperatures were estimated by linear regression using the four most extreme data points, either as high or low values and these regression lines were drawn in the figures.

## 3. Results and Discussion

**Figure 1:**
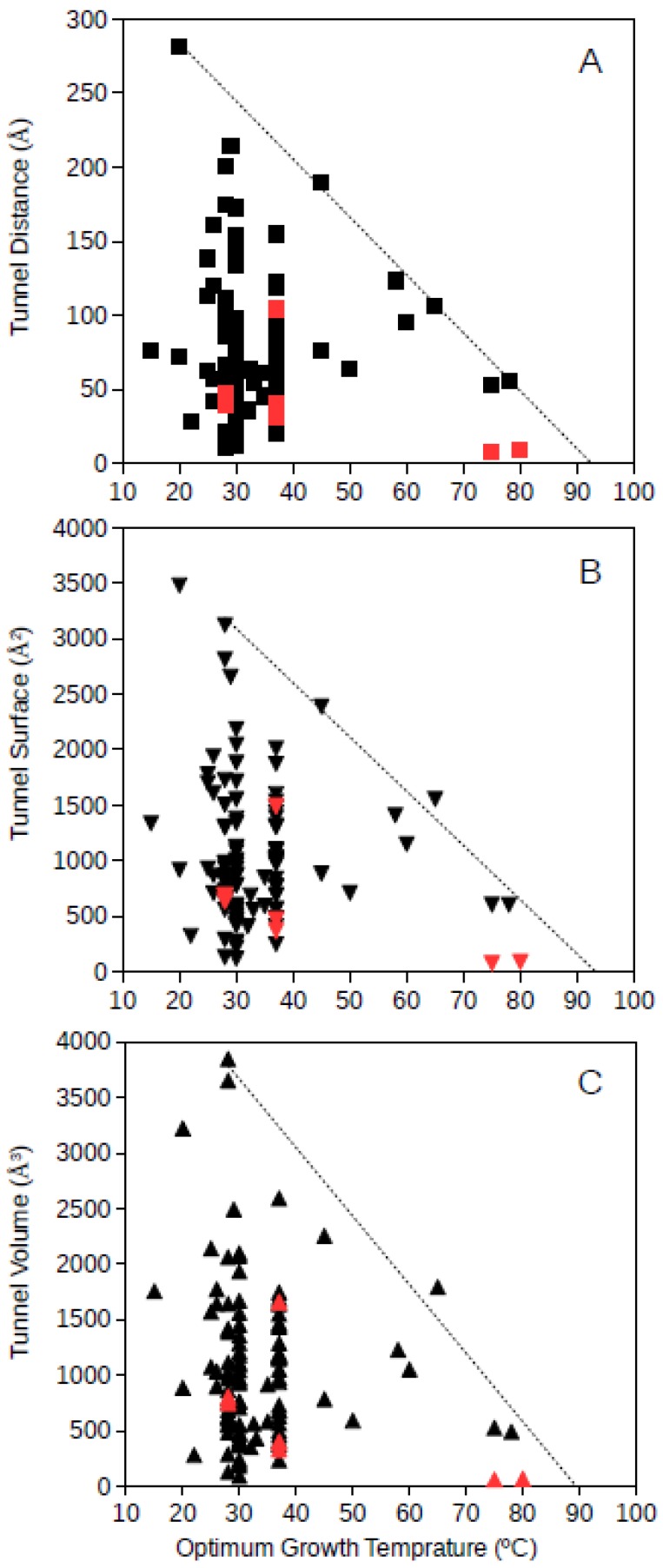
Tunnel distance (**A**), surface (**B**) and volume (**C**) (in Å) dimensions predicted for bacteria GDHs (Glutamate dehydrogenase) ranging in optimum growth temperature from 15 °C to 99 °C. Grey line represents a visualization of the upper limit for these dimensions at increasing temperatures. Black symbols correspond to predicted protein structures; red symbols correspond to resolved model structures from the Protein Data Bank.

**Figure 2:**
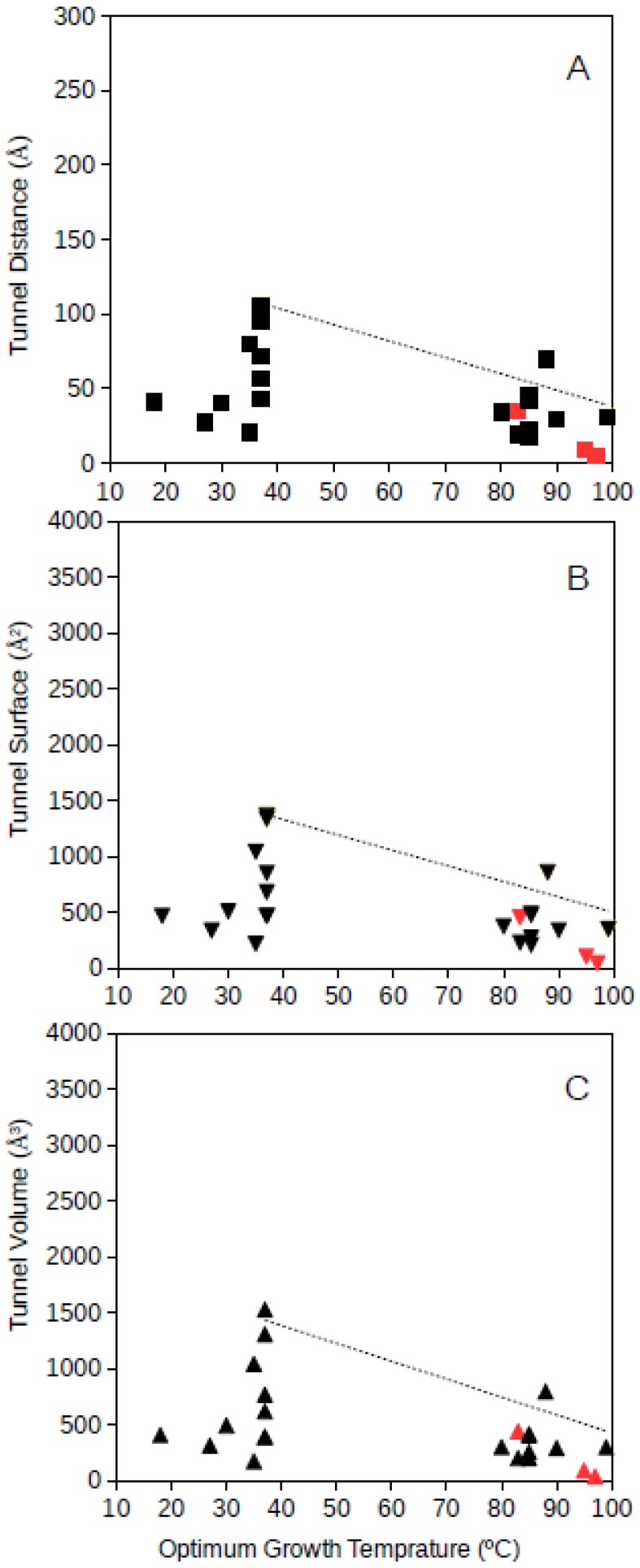
Tunnel distance (**A**), surface (**B**) and volume (**C**) (in Å) dimensions predicted for archaea GDHs ranging in optimum growth temperature from 15 °C to 99 °C. Grey line represents a visualization of the upper limit for these dimensions at increasing temperatures. Black symbols correspond to predicted protein structures; red symbols correspond to resolved model structures from the Protein Data Bank.

Tunnel distance, surface and volume estimates for each protein were plotted against the optimum growth temperature reported for the corresponding microorganism. For GDH, results showed that for proteins from microorganisms growing at high temperatures, the tunnel distance, surface and volume tend generally to decrease (Figure 1 and Figure 2). Approximated upper limit lines were drawn resulting in estimates of maximum tunnel distance, surface and volume values at 80 °C of 49 Å, 648 Å^2^, and 581 Å^3^, respectively, for bacterial GDH. The arqueal GDH data set suggested maximum tunnel distance, surface and volume values at 100 °C of 38 Å, 499 Å^2^, and 430 Å^3^, respectively. This decrease of channels and open spaces within the GDH molecules suggests that at increasing optimum growth temperatures these proteins get more compact, for example, increasing the number of interactions among closed amino acid residues and reducing the void spaces within the molecule. This is in agreement with previous understanding on the adaptations that proteins experience from hyperthermophiles [10] and specifically in GDH from hyperthermophiles [21,18]. The case of GDH, an enzyme in which molecular channels have not been reported to be functionally relevant, would show a decrease of the empty spaces within the protein 3D-structure to a minimum at high temperature. Thermophily imposes adaptive mechanisms, and so, the microorganisms adapted to grow at high temperatures present GDHs with a trend to minimum molecular channels.

**Figure 3:**
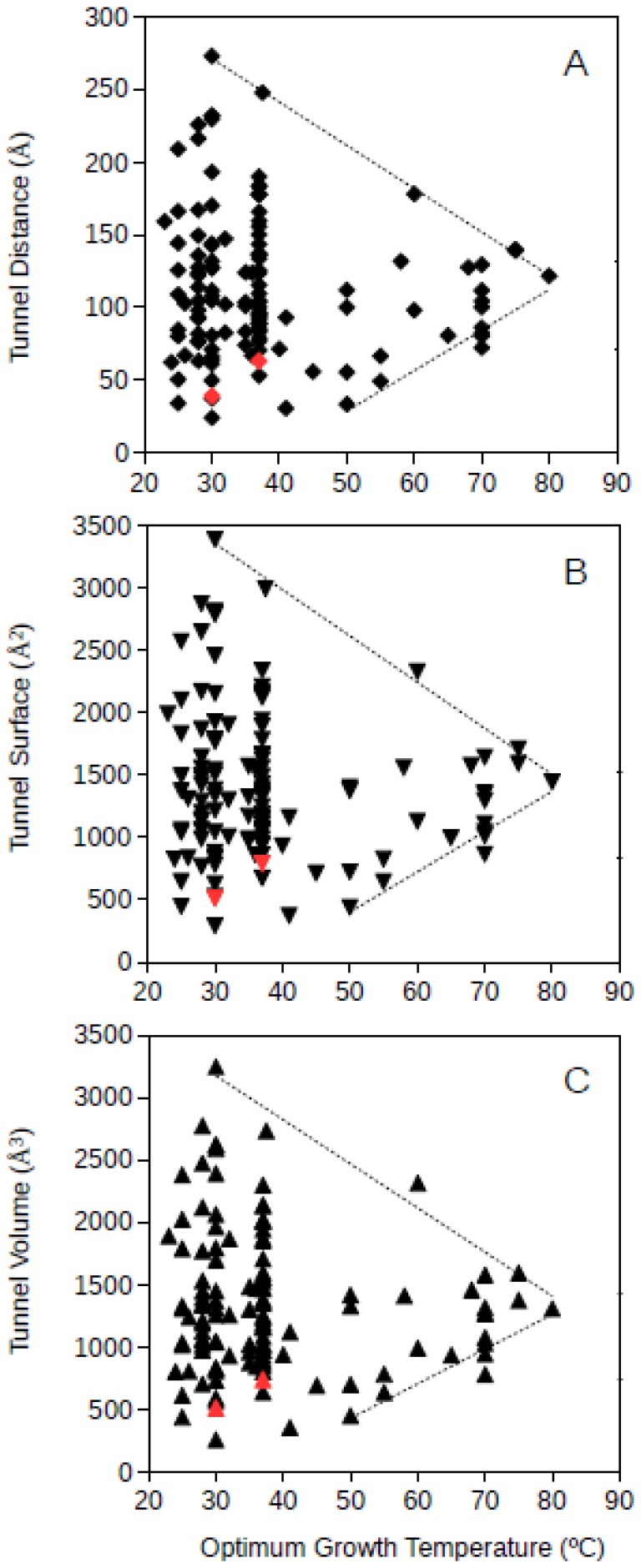
Tunnel distance (**A**), surface (**B**) and volume (**C**) (in Å) dimensions predicted for bacteria GPATs (glutamine phosphoribosylpyrophosphate amidotransferase) ranging in optimum growth temperature from 23 °C to 80 °C. Grey lines represent a visualization of the upper and lower limits for these dimensions at increasing temperatures. Black symbols correspond to predicted protein structures; red symbols correspond to resolved model structures from the Protein Data Bank.

Bacteria and archaea GPAT structures where analyzed separately. Bacterial GPAT tunnel distance, surface and volume where plotted against the optimum growth temperature of the corresponding bacteria (Figure 3). The results showed that at increasing optimum growth temperatures, the dimensions of the molecular tunnels points to a highly specific value narrowing down the large variability observed among mesophiles. The estimated values for bacterial GPAT were in the range 112–122 Å, 1368–1503 Å^2^, and 1262–1408 Å^3^ for the tunnel distance, surface and volume, respectively, (Figure 3) and were calculated at 80 °C from the regression lines drawn from the upper and lower limits of these dimensions. These estimates point towards the prediction of narrow ranges of dimensions for these tunnels in bacterial GPATs for microorganisms growing at high temperatures. These values obtained for bacterial GPAT are much higher than those observed for GDH (see above), suggesting that molecular tunnels are well maintained in GPATs from hyperthermophiles. These results also suggest an optimization of molecular channels within the bacterial GPAT molecules, because at increasing optimum growth temperatures the restrictive conditions push towards minimum unnecessary spaces within the molecule. Nevertheless, GPAT has been reported to require molecular tunnels within the enzyme, and so, the results from Figure 3 suggest that the required tunnels are maintained but superfluous spaces are compacted or filled by interactions among amino acid residues so that the protein is able to maintain its stability and function at the high temperatures required for growth. From this information, one can deduce that bacteria adapt to high temperatures by generating compact proteins, but the requirement for functionally relevant molecular tunnels is maintained. These results do not directly prove that molecular channels are important to maintain the metabolism in (hyper)thermophiles although they represent unimportant evidence on the relevance of these tunnels in hyperthermophiles. Maintaining the molecular tunnel dimensions, and so their functional features, at high optimum growth temperatures indicates that these tunnels are essential for thriving under those extreme conditions.

**Figure 4:**
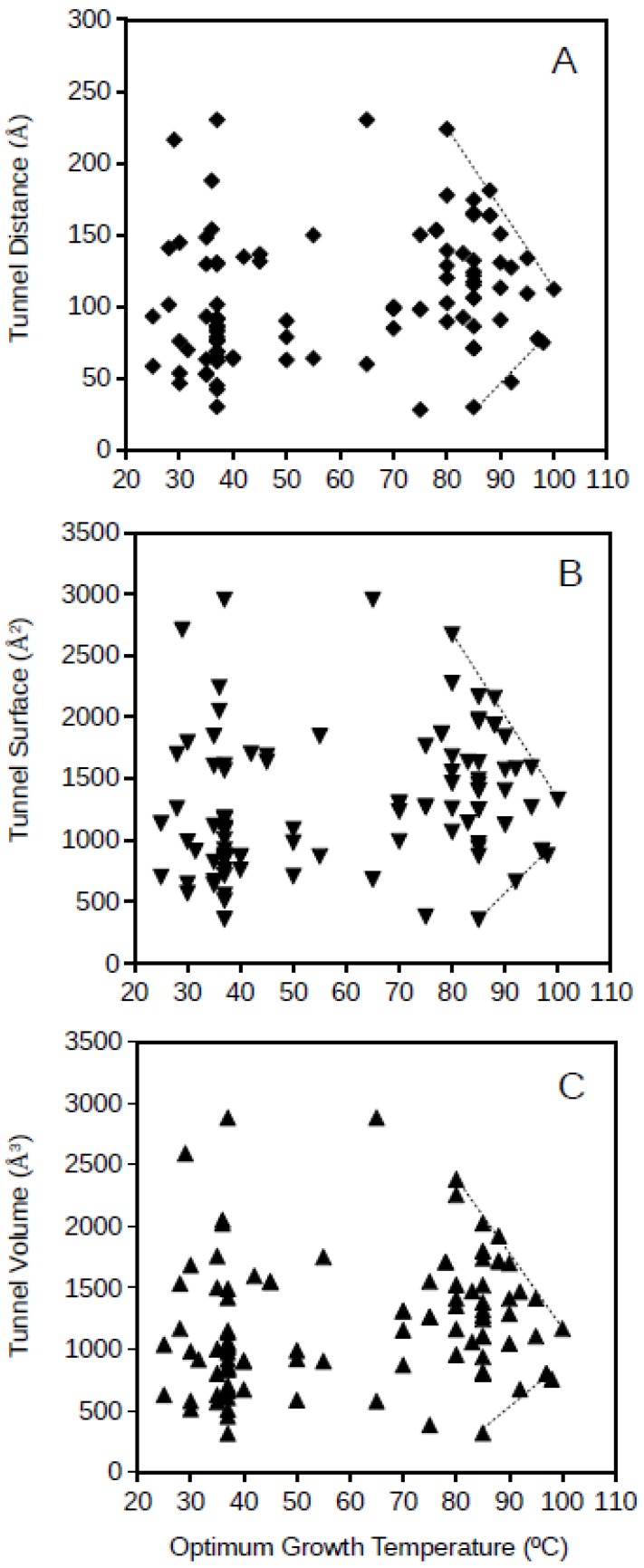
Tunnel distance (**A**), surface (**B**) and volume (**C**) (in Å) dimensions predicted for archaea GPATs ranging in optimum growth temperature from 25 °C to 100 °C. Grey lines represent a visualization of the upper and lower limits for these.

Archaeal GPAT tunnel distance, surface and volume plotted against the optimum growth temperature for the archaeon corresponding to each of the studied proteins is presented in Figure 4. Archaeal GPATs present apparently more disperse dimension values of molecular tunnels than bacterial GPATs, but a similar phenomenon to bacterial GPATs is observed for archaea, and the range of dimensions for the molecular tunnels in archaeal GPATs are limited for the highest studied optimum growth temperatures (above 80 °C; Figure 3). The upper and lower lines drawing these limits of tunnel dimensions point to the range of values of 85–113 Å, 998–1331 Å^2^, and 886–1260 Å^3^ for the distance, surface and volume, respectively, of archaeal GPAT molecular tunnels at an optimum growth temperature of 100 °C. This suggests that GPATs in hyperthermophiles also maintain the minimum required molecular tunnel dimensions in agreement to the above, when a scenario with similar dimensions for molecular tunnels for the bacterial GPAT was deduced. Interestingly, the dimensions of molecular tunnels for archaea and bacteria at the highest optimum growth temperatures are in agreement, showing similar values which suggest that these proteins are optimized about the channels and free spaces within the enzymes and reduced to the minimum functional required values. Because these minimum dimensions of molecular tunnels are maintained even at the most restrictive conditions imposed in hyperthermophiles and growth at high temperatures, the results suggest that these molecular tunnels are relevant for the functioning of the enzyme GPAT both in hyperthermophilic bacteria and archaea.

Because microorganisms are adapted to their environmental conditions, their enzymes need to reflect that adaptation. Thus, enzymes are optimized to best perform under the optimum growth temperature. The example of GDH shows a strategy to reduce voids within the protein structure so that it improves its thermostability. The case of GPAT suggests that molecular tunnels are required to maintain its functionality, but the dimensions of these tunnels must be within a narrow range to achieve optimum performance and stability at high temperatures.

The restrictions of molecular tunnel dimensions required in the microorganisms showing the highest optimum growth temperatures represent a requirement to build functional enzymes such as GPAT in hyperthermophiles. Establishing the requirement of molecular tunnels in specific enzymes of hyperthermophilic archaea and bacteria is a step forward, contributing to understand the mechanisms of thermophily in relationship to protein function and structure. Herein, it has been shown that molecular tunnels are required at high temperatures. Future studies might start to delve deeper into the consequences for this requirement. Although the high thermal stability of proteins (i.e., and enzymes) from hyperthermophiles has been clearly demonstrated [22,10], the capability of hyperthermophiles to maintain the stability of universal, thermolabile low-molecular weight biomolecules (e.g., NADH, NADPH, ATP, pyridoxal phosphate, etc.) remains to be understood. One of the mechanisms most reported for hyperthermophiles to be able to thrive at high temperatures using these thermolabile low-molecular weight biomolecules has been the existence of molecular channels or tunnels that could directly transfer these labile substrates to the active sites of the enzymes maintaining longer stability [11,12,15]. Herein, the requirement of molecular tunnels of specific dimensions is shown for GPAT from hyperthermophiles. This information brings up the potential for the relationship of these molecular tunnels to the maintenance of thermostability and the transference of thermolabile low molecular weight biomolecules to make possible an effective metabolism at high temperatures.

## Supplementary Materials

The following are available online at http://www.mdpi.com/2076-2607/6/4/109/s1.
