# Supplementary material for: Molecular Tunnels in Enzymes and Thermophily: A Case Study on the Relationship to Growth Temperature
Source: Microorganisms. 2018 Oct 20;6(4):109. doi: 10.3390/microorganisms6040109 (PMC6313320; doi:10.3390/microorganisms6040109)
Supplement: Supplementary file 1 [file microorganisms-06-00109-s001.pdf]

GDH. List of genes and optimum growth temperatures

Opt. Temp. Growth (ºC)	Accession_number_and_Species
22	ELS30268.1 [Pseudanabaena_biceps_PCC_7429]
37	EGJ70233.1 [Bacteroides_coprosuis_DSM_18011]
30	ALS79895.1 [Planococcus_kocurii]
45	AEW04907.1 [Sulfobacillus_acidophilus_DSM_10332]
37	AEE13276.1 [Porphyromonas_asaccharolytica_DSM_20707]
37	EMD99258.1 [Pseudomonas_stutzeri_NF13]
30	EKU29406.1 [Alcaligenes_sp._HPC1271]
37	EIA16798.1 [Clostridium_perfringens_F262]
37	EHA17544.1 [Halomonas_sp._HAL1]
37	EGE27438.1 [Moraxella_catarrhalis_O35E]
37	EGE26670.1 [Moraxella_catarrhalis_101P30B1]
37	EGA38201.1 [Salmonella_enterica_subsp._enterica_serovar_Montevideo_str._IA_2010008282]
37	AUY29140.1 [Escherichia_coli]
30	ASV30406.1 [Maribacter_cobaltidurans]
28	ATL83975.1 [Streptomyces_malaysiensis]
26	AFV86422.1 [Alteromonas_mediterranea_DE1]
50	SMC01596.1 [Sulfobacillus_thermosulfidooxidans_DSM_9293]
37	SMB92486.1 [Peptoniphilus_asaccharolyticus_DSM_20463]
28	OOP61299.1 [Arthrobacter_sp._SRS-W-1-2016]
30	AND41967.1 [Bacillus_oceanisediminis_2691]
28	KXA34217.1 [Staphylococcus_aureus]
37	AFX90100.1 [Helicobacter_pylori_Aklavik86]
25	BAP84658.1 [Lactobacillus_hokkaidonensis_JCM_18461]
28	AFM59527.1 [Enterobacter_cloacae_subsp._dissolvens_SDM]
37	ADY36188.1 [Bacteroides_salanitronis_DSM_18170]
37	AGW84240.1 [Bifidobacterium_animalis_subsp._lactis_ATCC_27673]
78	KHC95959.1 [Thermotoga_sp._Xyl54]-
30	KAJ28402.1 [Pseudomonas_aeruginosa_M10]
37	AFU41346.1 [Propionibacterium_acnes_C1]
30	AFU45269.1 [Acidovorax_sp._KKS102]
26	ABI77516.1 [Hyphomonas_neptunium_ATCC_15444]
25	EAQ42780.1 [Polaribacter_sp._MED152]
65	AEV15549.1 [Thermus_sp._CCB_US3_UF1]
28	AFK63375.1 [Advenella_kashmirensis_WT001]
30	EQM83169.1 [Stenotrophomonas_maltophilia_MF89]
37	EMG30292.1 [Campylobacter_showae_CC57C]
28	EME69651.1 [Magnetospirillum_caucaseum]
37	EKT62111.1 [Providencia_alcalifaciens_Dmel2]
30	EGU00928.1 [Acinetobacter_baumannii_ABNIH3]
30	EGO65932.1 [Acetonema_longum_DSM_6540]
37	EGF49896.1 [Actinomyces_sp._oral_taxon_170_str._F0386]
30	EEL84441.1 [Bacillus_cereus_AH1272]
30	ELK41699.1 [Brevibacillus_agri_BAB-2500]
26	AFV85481.1 [Alteromonas_mediterranea_DE1]
28	ORL45099.1 [Zunongwangia_atlantica_22II14-10F7]
37	ALU83768.1 [Listeria_monocytogenes]
20	AMP97068.1 [Pedobacter_cryoconitis]
37	KXB81812.1 [Varibaculum_cambriense]
30	ALV09269.1 [Roseateles_depolymerans]

30	ALO46674.1 [Pseudohongiella_spirulinae]
30	CUH53241.1 [Shimia_marina]
28	KRG28254.1 [Salegentibacter_mishustinae]
30	AIE32843.1 [Bacillus_thuringiensis_serovar_kurstaki_str._HD-1]
26	CEM57971.1 [Xanthomonas_campestris_pv._campestris]
30	CAP42835.1 [Bordetella_petrii]
28	KHE92493.1 [Candidatus_Scalindua_brodae]
30	KGH26017.1 [Comamonas_testosteroni]
37	AHG81894.1 [Bibersteinia_trehalosi_USDA-ARS-USMARC-188]
37	AHG79658.1 [Mannheimia_varigena_USDA-ARS-USMARC-1388]
30	KFX63213.1 [Burkholderia_sp._K24]
30	ETT63245.1 [Paenibacillus_sp._FSL_R7-277]
29	EPE93997.1 [Rhizobium_grahamii_CCGE_502]
37	EPF23053.1 [Klebsiella_pneumoniae_subsp._pneumoniae_B5055]
28	EOS93342.1 [Erwinia_tracheiphila_PSU-1]
30	EPC65060.1 [Lactobacillus_paracasei_subsp._tolerans_Lpl14]
37	EOR21003.1 [Clostridium_sartagoforme_AAU1]
30	EON72493.1 [Lysinibacillus_sphaericus_OT4b.31]
37	AGH38306.1 [Bibersteinia_trehalosi_USDA-ARS-USMARC-192]
30	ENO96228.1 [Thauera_phenylacetica_B4P]
30	EMY34539.1 [Arthrobacter_crystallopoietes_BAB-32]
30	EMT90349.1 [Acinetobacter_baumannii_ABNIH5]
30	EMF06805.1 [Serratia_marcescens_VGH107]
37	EMC47454.1 [Streptococcus_mutans_SA38]
37	ELV06143.1 [Brachyspira_hampsonii_30599]
28	ELV07312.1 [Wohlfahrtiimonas_chitiniclastica_SH04]
32	ELP52983.1 [Microcystis_aeruginosa_TAIHU98]
30	ELK46429.1 [Halobacillus_sp._BAB-2008]
28	EKT78693.1 [Rhodococcus_opacus_M213]
75	EKF50234.1 [Thermosipho_africanus_H17ap60334]
35	EKE32837.1 [Salimicrobium_jeotgali]
28	EKF36941.1 [Bacillus_xiamenensis]
28	EHJ96541.1 [Agrobacterium_tumefaciens_5A]
28	EGD54612.1 [Gordonia_neofelifaecis_NRRL_B-59395]
30	ADM71247.1 [Paenibacillus_polymyxa_E681]
28	WP_014332063.1 [Sinorhizobium_fredii]
30	WP_005000751.1 [Nitrococcus_mobilis]
30	PJM66389.1 [Bacillus_subtilis]
28	AKX57894.1 [Oblitimonas_alkaliphila]
28	PBI84830.1 [Variovorax_boronicumulans]
37	OZS77933.1 [Planococcaceae_bacterium_VT-49_Tetzosporium_himinis]
30	OZQ78918.1 [Paenibacillus_odorifer]
37	OZG65257.1 [Bifidobacterium_eulemuris]
37	OYN34808.1 [Enterococcus_faecalis]
28	OXS15995.1 [Zobellella_denitrificans]
99	WP_011012748.1 [Pyrococcus_furiosus]
85	BAN90344.1 [Aeropyrum_camini_SY1_=_JCM_12091]
90	BAA80383.2 [Aeropyrum_pernix_K1]
37	BAI60865.1 [Methanocella_paludicola_SANAE]
37	KQC15291.1 [Methanosaeta_sp._SDB]
27	KPQ41220.1 [Candidatus_Methanoperedens_sp._BLZ1]
37	AIS31989.1 [Methanobacterium_formicicum]

37	AAM30053.1 [Methanosarcina_mazei_Go1]
35	PAV08686.1 [Methanocorpusculum_parvum]
35	AKJ39708.1 [Methanosarcina_barkeri_CM1]
30	KGK99756.1 [Methanococcoides_methylutens]
37	AUB59784.1 [Methanobacterium_subterraneum]
30	KQC12212.1 [Desulfuromonas_sp._SDB]
33	KQC10805.1 [Candidatus_Cloacimonas_sp._SDB]
37	AEE91450.1 [Tepidanaerobacter_acetatoxydans_Re1]
30	KIX15625.1 [Dethiosulfatarculus_sandiegensis]
20	EEB84258.1 [Roseobacter_sp._GAI101]
28	EED35449.1 [Luminiphilus_syltensis_NOR5-1B]
25	EDZ42865.1 [Rhodobacteraceae_bacterium_HTCC2083]
28	AUZ85152.1 [Methylophaga_nitratireducenticrescens]
35	GAP44769.1 [Lentimicrobium_saccharophilum]
45	EEX11196.1 [Silicibacter_lacuscaerulensis_ITI-1157]
15	EDZ62302.1 [Sulfurimonas_gotlandica_GD1]
58	KIE58786.1 [Methylacidiphilum_kamchatkense_Kam1]
28	AFI85374.1 [Methylophaga_nitratireducenticrescens]
32.5	AHB49186.1 [Hyphomicrobium_nitrativorans_NL23]
60	ACD83816.1 [Methylacidiphilum_infernorum_V4]
18	AFV23154.1 [Methanolobus_psychrophilus_R15]
37	AAM06542.1 [Methanosarcina_acetivorans_C2A]
85	BAD85620.1 [Thermococcus_kodakarensis_KOD1]
85	AAK53112.1 [Thermococcus_waiotapuensis]
83	EHR77478.1 [Thermococcus_litoralis_DSM_5473]
80	ASJ02396.1 [Thermococcus_profundus]
80	BAA28943.1 [Thermococcus_profundus]
85	AMQ19106.1 [Thermococcus_peptonophilus]
88	WP_088866669.1 [Thermococcus_radiotolerans]

GDH. List of genes with a resolved structure at RCSB Protein Databank

Opt. Temp. Growth (ºC)	Accession_number_PDB	Species
37	1AUP	Clostridium symbiosum
80	1B26	Thermotoga maritima
83	1BVU	Thermococcus litorlis
97	1GTM	Pyrococcus furiosus
95	1V9L	Pyrobaculum islandicum
37	2YFQ	Peptoniphilus asaccharolyticus
75	3AOE	Thermus thermophilus
37	4FCC	Escherichia coli
28	4XGI	Burkholderia thailandensis
28	5IJZ	Corynebacterium glutamicum

GPAT. Bacteria. List of genes and optimum growth temperatures

Opt. Temp. Growth (ºC)	Accession_number_and_Species
25	ELS32782.1 [Pseudanabaena_biceps_PCC_7429]
37	EIC03023.1 [Treponema_saccharophilum_DSM_2985]
30	ATG90710.1 [Methylomonas_koyamae]
35	ADO76313.1 [Halanaerobium_praevalens_DSM_2228]
30	EJZ59104.1 [Pseudomonas_fluorescens_R124]
60	EHO40009.1 [Caldithrix_abyssi_DSM_13497]
37	EFQ23609.1 [Aminomonas_paucivorans_DSM_12260]
37	ADL13794.1 [Acetohalobium_arabaticum_DSM_5501]
25	EIJ33952.1 [Thiothrix_nivea_DSM_5205]
30	ADP97585.1 _[Marinobacter_adhaerens_HP15]
37	EHY31206.1 [Sutterella_parvirubra_YIT_11816]
37	EFF63510.1 [Turicibacter_sanguinis_PC909]
32	APV44660.1 [Dehalogenimonas_formicexedens]
80	AEH50552.1 [Pseudothermotoga_thermarum_DSM_5069]
35	ADK82223.1 [Sediminispirochaeta_smaragdinae_DSM_11293]
41	EAY55918.1 [Leptospirillum_rubarum]
30	ALS79409.1 [Planococcus_kocurii]
55	ADR18966.1 [Calditerrivibrio_nitroreducens_DSM_19672]
30	EJZ60418.1 [Pseudomonas_fluorescens_R124]
30	EHQ07781.1 [Leptonema_illini_DSM_21528]
37	AEE16096.1 [Treponema_brennaborense_DSM_12168]
30	AEC02086.1 [Sphaerochaeta_coccoides_DSM_17374]
37	AEB13779.1 [Treponema_succinifaciens_DSM_2489]
70	ADY73919.1 [Desulfurobacterium_thermolithotrophum_DSM_11699]
37	ADY37927.1 [Bacteroides_salanitronis_DSM_18170]
55	AEA33247.1 [Hippea_maritima_DSM_10411]
37	ADV45935.1 [Nitratifractor_salsuginis_DSM_16511]
28	ADU49710.1 [Intrasporangium_calvum_DSM_43043]
30	KAJ26249.1 [Pseudomonas_aeruginosa_M10]
26	ABI75600.1 [Hyphomonas_neptunium_ATCC_15444]
45	ADI14672.1 [Truepera_radiovictrix_DSM_17093]
28	ADH69776.1 [Nocardiopsis_dassonvillei_subsp._dassonvillei_DSM_43111]
25	ADG92116.1 [Arcobacter_nitrofigilis_DSM_7299]
28	ADN76808.1 [Ferrimonas_balearica_DSM_9799]
37	ERH14168.1 [Actinomyces_graevenitzii_F0530]
50	AFZ36656.1 [Stanieria_cyanosphaera_PCC_7437]
25	AFZ13352.1 [Crinalium_epipsammum_PCC_9333]
25	AFZ09650.1 [Oscillatoria_nigro-viridis_PCC_7112]
25	AFY90730.1 [Chroococcidiopsis_thermalis_PCC_7203]
25	AFZ60793.1 [Anabaena_cylindrica_PCC_7122]
28	AEH89674.1 [Mesorhizobium_opportunistum_WSM2075]
50	AEE96815.1 [Mahella_australiensis_50-1_BON]
37.5	EFY07712.1 [Succinatimonas_hippei_YIT_12066]
37	EFY04338.1 [Phascolarctobacterium_succinatutens_YIT_12067]
35	EFU92919.1 [Enterococcus_faecalis_TX0309A]
37	EFU88199.1 [Enterococcus_faecalis_TX0309B]
37	EFS06536.1 [Enterococcus_faecium_TX0133a04]
37	EFQ03885.1 [Megasphaera_micronuciformis_F0359]
37	EFO60743.1 [Escherichia_coli_MS_145-7]

37 EFK72573.1 [Escherichia_coli_MS_78-1]
37 EFK50290.1 [Escherichia_coli_MS_107-1]
25 ADR32891.1 [Sulfuricurvum_kujiense_DSM_16994]
68 ADL08250.1 [Thermosediminibacter_oceani_DSM_16646]
30 EHP88642.1 [Geobacter_metallireducens_RCH3]
30 EHO50524.1 [Lactobacillus_kisonensis_F0435]
37 EHM54848.1 [Cardiobacterium_valvarum_F0432]
37 EHM42883.1 [Anaeroglobus_geminatus_F0357]
35 EHB50180.1 [Paenibacillus_lactis_154]
37 EGG51651.1 [Parasutterella_excrementihominis_YIT_11859]
65 EGD51504.1 [Thermoanaerobacter_ethanolicus_JW_200]
37 EGA37672.1 [Salmonella_enterica_subsp._enterica_serovar_Montevideo_str._IA_2010008282]
37 EFN39130.1 [Escherichia_coli_W]
30 EFM10745.1 [Paenibacillus_curdlanolyticus_YK9]
30 EED68647.1 [Comamonas_testosteroni_KF-1]
28 EDT12485.1 [Paraburkholderia_graminis_C4D1M]
30 ASV32642.1 [Maribacter_cobaltidurans]
30 ASV30329.1 [Maribacter_cobaltidurans]
28 ATL84365.1 [Streptomyces_malaysiensis]
28 ALL67914.1 [Paraburkholderia_caribensis_MBA4]
28 OXC74793.1 [Caballeronia_sordidicola]
32 ASC72192.1 [Halomicronema_hongdechloris_C2206]
28 OTP78821.1 [Caballeronia_sordidicola]
37 ORI05316.1 [Staphylococcus_sciuri]
37 AMY87605.1 [Salmonella_enterica_enterica_Anatum_str._USDA-ARS-USMARC-1765]
28 ADV68409.1 [Deinococcus_maricopensis_DSM_21211]
30 ACL05170.1 [Desulfatibacillum_alkenivorans_AK-01]
37 AMZ11208.1 [Salmonella_enterica_enterica_Anatum_str._USDA-ARS-USMARC-1728]
30 AND37944.1 [Bacillus_oceanisediminis_2691]
37 ALU82184.1 [Listeria_monocytogenes]
37 KXB88689.1 [Veillonella_dispar]
37 KXB85585.1 [Veillonella_parvula]
37 KXB82054.1 [Varibaculum_cambriense]
37 KXB73216.1 [Peptostreptococcus_anaerobius]
30 KXB45491.1 [Citrobacter_koseri]
37 KXB61404.1 [Leptotrichia_wadei]
37 KXA67059.1 [Cutibacterium_avidum]
37 KXA64523.1 [Veillonella_atypica]
30 KXA06459.1 [Citrobacter_koseri]
37 KXA03004.1 [Clostridium_perfringens]
40 KWZ85402.1 [Bacillus_coagulans]
37 KWZ74493.1 [Actinomyces_neuii]
28 GAE58782.1 [Xanthomonas_arboricola_pv._pruni_MAFF_301427]
41 EIM29599.1 [Microvirga_lotononidis]
60 ADR36146.1 [Oceanithermus_profundus_DSM_14977]
28 AKB06715.1 [Vibrio_cholerae]
30 AJJ35492.1 [Yersinia_kristensenii]
37 AJI69363.1 [Francisella_tularensis_subsp._tularensis_SCHU_S4]
37 AJI66878.1 [Francisella_tularensis_subsp._holarctica]
23 EGW22096.1 [Methylobacter_tundripaludum_SV96]
70 EIW00571.1 [Thermoanaerobacter_siderophilus_SR4]
28 EHY89476.1 [Saccharomonospora_azurea_NA-128]

28 EHR63782.1 [Saccharomonospora_cyanea_NA-134]
35 EHQ87806.1 [Desulfosporosinus_youngiae_DSM_17734]
25 EHQ15578.1 [Shewanella_baltica_OS183]
36 KIY07823.1 [Burkholderia_mallei]
30 AHB10872.1 [Zymomonas_mobilis_subsp._mobilis_str._CP4_=_NRRL_B-14023]
37 ADW17682.1 [Desulfobulbus_propionicus_DSM_2032]
58 AER66880.1 [Thermovirga_lienii_DSM_17291]
70 AEH44909.1 [Thermodesulfatator_indicus_DSM_15286]
37 AEB09977.1 [Desulfobacca_acetoxidans_DSM_11109]
50 AEE14257.1 [Thermodesulfobium_narugense_DSM_14796]
70 AEB11312.1 [Marinithermus_hydrothermalis_DSM_14884]
28 ADY56644.1 [Syntrophobotulus_glycolicus_DSM_8271]
30 ADX68316.1 [Weeksella_virosa_DSM_16922]
75 ADU51534.1 [Thermaerobacter_marianensis_DSM_12885]
26 AIT05447.1 [Sphingomonas_taxi]
24 ADN09900.1 [Sulfurimonas_autotrophica_DSM_16294]
50 ADH62957.1 [Meiothermus_silvanus_DSM_9946]
28 EFH87731.1 [Ktedonobacter_racemifer_DSM_44963]
30 ADY25367.1 [Deinococcus_proteolyticus_MRP]
70 ADQ40935.1 [Caldicellulosiruptor_kristjanssonii_I77R1B]
37 ADY34043.1 [Odoribacter_splanchnicus_DSM_20712]
28 EUJ10237.1 [Methylophilaceae_bacterium_11]
75 AFH38428.1 [Thermus_thermophilus_JL-18]
70 AFV75405.1 [Thermus_oshimai_JL-2]
70 EED11250.1 [Thermus_aquaticus_Y51MC23]
70 APD08922.1 [Thermus_brockianus]
36 AJF02165.1 [Mycobacterium_tuberculosis_H37RvSiena]
32 BAZ93959.1[Thiohalobacter_thiocyanaticus]

GPAT. List of genes with a resolved structure at RCSB Protein Databank

Opt. Temp. Growth (ºC)	Accession_number_PDB	Species
30	1AO0	Bacillus subtilis
37	1ECF	Escherichia coli

GPAT. Archaea. List of genes and optimum growth temperatures

Opt. Temp. Growth (ºC)	Accession_number_and_Species
37	EJG08245.1 [Methanofollis_liminatans_DSM_4140]
30	EHQ35559.1 [Methanoplanus_limicola_DSM_2279]
37	ADE35893.1 [Methanohalophilus_mahii_DSM_5219]
90	ADN49979.1 [Vulcanisaeta_distributa_DSM_14429]
92	ADM27115.1 [Ignisphaera_aggregans_DSM_17230]
83	ADP77180.1 [Methanothermus_fervidus_DSM_2088]
75	AEA47242.1 [Archaeoglobus_veneficus_SNP6]
70	EHP85562.1 [Methanotorris_formicicus_Mc-S-70]
90	AFL66391.1 [Desulfurococcus_amylolyticus_DSM_16532]
92	ADM27114.1 [Ignisphaera_aggregans_DSM_17230]
37	ADN37401.1 [Methanolacinia_petrolearia_DSM_11571]
45	AEH61214.1 [Methanosalsum_zhilinae_DSM_4017]
97	AAL80278.1 [Pyrococcus_furiosus_DSM_3638]
25	ETA67047.1 [Methanolobus_tindarius_DSM_2278]
29	EHP68719.1 [Metallosphaera_yellowstonensis_MK1]
28	ACL15768.1 [Methanosphaerula_palustris_E1-9c]
75	ADX82816.1 [Sulfolobus_islandicus_HVE10/4]
36	ABS54619.1 [Methanoregula_boonei_6A8]
75	ADX82815.1 [Sulfolobus_islandicus_HVE10/4]
37	ADE04206.1 [Haloferax_volcanii_DS2]
37	ADD04744.1 [Natrialba_magadii_ATCC_43099]
40	KQB36740.1 [Acidiplasma_aeolicum]
55	KQB35509.1 [Acidiplasma_cupricumulans]
50	ADI73116.1 [Methanohalobium_evestigatum_Z-7303]
37	ABX02336.1 [Methanococcus_maripaludis_C6]
70	ADD09196.1 [Aciduliprofundum_boonei_T469]
85	ADC65987.1 [Ferroglobus_placidus_DSM_10642]
37	ADB61534.1 [Haloterrigena_turkmenica_DSM_5511]
80	ACX72222.1 [Methanocaldococcus_vulcanius_M7]
85	ACV24300.1 [Methanocaldococcus_fervens_AG86]
30	ACM57065.1 [Halorubrum_lacusprofundi_ATCC_49239]
35	ABR54375.1 [Methanococcus_vannielii_SB]
37	ABO34754.1 [Methanococcus_maripaludis_C5]
28	ABN56162.1 [Methanoculleus_marisnigri_JR1]
90	ADN49980.1 [Vulcanisaeta_distributa_DSM_14429]
85	ADB58053.1 [Archaeoglobus_profundus_DSM_5631]
35	ADI36027.1 [Methanococcus_voltae_A3]
85	ADG13026.1 [Methanocaldococcus_infernus_ME]
70	ACX91911.1 [Sulfolobus_solfataricus_98/2]
37	ABR65465.1 [Methanococcus_maripaludis_C7]
40	ABR55881.1 [Methanococcus_aeolicus_Nankai-3]
90	CCE69597.1 [Pyrococcus_abyssi_GE5]
30	PAV13552.1 [Methanosarcina_spelaei]
35	PAV08642.1 [Methanocorpusculum_parvum]
35	PAV08637.1 [Methanocorpusculum_parvum]
37	PAV07091.1 [Methanosphaera_cuniculi]
37	PAV06251.1 [Methanobacterium_bryantii]
31.5	AGB01133.1 [Methanoregula_formicica_SMSP]
85	ACB40826.1 [Pyrobaculum_neutrophilum_V24Sta]

85	ACB40819.1 [Pyrobaculum_neutrophilum_V24Sta]
85	WP_088853670.1 [Thermococcus_pacificus]
75	ADX85444.1 _[Sulfolobus_islandicus_REY15A]
80	ARM76262.1 [Acidianus_manzaensis]
80	ARM76261.1 [Acidianus_manzaensis]
100	AMM53159.1 [Pyrococcus_kukulkanii]
36	BAI62354.1 [Methanocella_paludicola_SANAE]
80	BAD84400.1 [Thermococcus_kodakarensis_KOD1]
70	SAI84180.1 [Sulfolobus_solfataricus]
85	WP_062387766.1 [Thermococcus_peptonophilus]
88	WP_062372122.1 [Thermococcus_guaymasensis]
85	ALM76321.1 [Thermococcus_barophilus]
85	AMQ18195.1 [Thermococcus_peptonophilus]
88	AJC71945.1 [Thermococcus_guaymasensis_DSM_11113]
78	KUK18077.1 [Thermococcus_sibiricus]
80	WP_058939691.1 [Thermococcus_celericrescens]
85	WP_056934730.1 [Thermococcus_barophilus]
50	AFD01075.1 [Methanocella_conradii_HZ254]
40	KPV46378.1 [Acidiplasma_aeolicum]
80	AFL94998.1 [Thermococcus_cleftensis]
80	AIF68565.1 [Palaeococcus_pacificus_DY20341]
85	WP_050001967.1 [Thermococcus_eurythermalis]
85	AIU68983.1 [Thermococcus_eurythermalis]
37	ADQ66777.1 [Halogeometricum_borinquense_DSM_11551]
88	WP_048811074.1 [Thermococcus_gammatolerans]
95	WP_048053066.1 [Pyrococcus_horikoshii]
37	AGB31296.1 [Natrinema_pellirubrum_DSM_15624]
37	AGB48961.1 [Methanomethylovorans_hollandica_DSM_15978]
83	EHR78953.1 [Thermococcus_litoralis_DSM_5473]
65	AEH06237.1 [Methanothermococcus_okinawensis_IH1]
35	AKB54280.1 [Methanosarcina_barkeri_MS]
35	AKB57641.1 [Methanosarcina_barkeri_227]
37	AKB44441.1 [Methanosarcina_vacuolata_Z-761]
30	AKB84409.1 [Methanococcoides_methylutens_MM1]
35	AKB82028.1 [Methanosarcina_barkeri_3]
37	AKB79529.1 [Methanosarcina_horonobensis_HB-1_=_JCM_15518]
25	AKB75657.1 [Methanosarcina_lacustris_Z-7289]
37	AKB70852.1 [Methanosarcina_mazei_C16]
37	AKB67501.1 [Methanosarcina_mazei_LYC]
37	AKB64156.1 [Methanosarcina_mazei_S-6]
37	AKB60899.1 [Methanosarcina_mazei_SarPi]
37	AKB39939.1 [Methanosarcina_mazei_WWM610]
37	AKB37833.1 [Methanosarcina_siciliae_C2J]
37	AKB27838.1 [Methanosarcina_siciliae_T4/M]
37	AKB31762.1 [Methanosarcina_siciliae_HI350]
50	AKB16521.1 [Methanosarcina_thermophila_CHTI-55]
50	AKB12858.1 [Methanosarcina_thermophila_TM-1]
45	CAJ35223.1 [Methanocella_arvoryzae_MRE50]
37	AIM28104.1 [Metallosphaera_sedula]
85	AIG97747.1 [Archaeoglobus_fulgidus_DSM_8774]
42	AIC14836.1 [Nitrososphaera_viennensis_EN76]
98	AEH23782.1 [Pyrococcus_yayanosii_CH1]

85 ADT85007.1 [Thermococcus_barophilus_MP]
97 AFN04422.1 [Pyrococcus_furiosus_COM1]
78 ACS89134.1 [Thermococcus_sibiricus_MM_739]
55 AAT43254.1 [Picrophilus_torridus_DSM_9790]
37 AAM30037.1 [Methanosarcina_mazei_Go1]
65 ABP96121.1 [Metallosphaera_sedula_DSM_5348]
90 ABU82082.1 [Ignicoccus_hospitalis_KIN4/I]
95 ABP51736.1 [Pyrobaculum_arsenaticum_DSM_13514]
